# Magnitude of substance use and associated factors among Wallaga University undergraduate students, Western Ethiopia, 2024

**DOI:** 10.3389/fpubh.2025.1519425

**Published:** 2025-03-25

**Authors:** Keno Firezer Belay, Eba Abdissa Golja, Adisu Ewunetu Desisa, Gelane Gurmu Gobena, Desalegn Biru Bulbula, Lalisa Mekonnen Moti, Keneni Efrem Dibbisa, Worku Fikadu, Getahun Fetensa

**Affiliations:** ^1^School of Public Health, Institute of Health Science, Wallaga University, Nekemte, Ethiopia; ^2^Gimbi General Hospital, Oromia Regional Health Bureau, Gimbi, Ethiopia; ^3^Department of Anthropology, Faculty of Social Science and Humanities, Wallaga University, Nekemte, Ethiopia; ^4^West Wallaga Zone Health Department, Oromia Regional Health Bureau, Gimbi, Ethiopia; ^5^Department of Health Behavior and Society, Faculty of Public Health, Jimma Medical Center, Jimma University, Jimma, Ethiopia

**Keywords:** substance use, Wallaga University, undergraduate students, factors, Ethiopia

## Abstract

**Background:**

Substance use among undergraduate university students has been recognized as a global public health issue; however, little attention is given to addressing this issue. Limited research has been conducted on substance use and its associated factors in the western part of Ethiopia, including our study area, with the same study population. Therefore, this study aimed to assess the magnitude of substance use and its associated factors among undergraduate students at Wallaga University in Western Ethiopia in 2024.

**Materials and methods:**

An institutional-based cross-sectional study was conducted involving 674 undergraduate students at Wallaga University from 25 March 2024 to 2 May 2024. Multistage cluster sampling was used for the sampling procedures, and data were collected using a self-administered questionnaire developed using the World Health Organization (WHO) ASSIST V3.0 tool. The collected data were entered into EpiData v.4.6 and analyzed using Statistical Package for the Social Sciences (SPSS) v.26. Variables with a *p*-value of <0.25 in the bivariate analysis were entered into multivariable logistic regression. Adjusted odds ratios and the corresponding 95% confidence intervals (CIs) were used to quantify the degree of association. A *p*-value of ≤0.05 was considered statistically significant in the final model.

**Results:**

A total of 674 students were included in the analysis, achieving a response rate of 94%. The mean age of the study participants was 22.66 ± 2.21 years (SD). The magnitude of current substance use was 29.5% (29.5, 95%CI: 25.96–33.04%). The current use of alcohol, khat, and tobacco was reported by 145 (22.8%) participants, 94 (14.8%) participants, and 16 (2.5%) participants, respectively. Factors such as being male (AOR =1.95, 95%CI: 1.27–2.78), having pocket money greater than 1,000 ETB (AOR = 2.27, 95%CI: 1.20–4.28), being a natural science student (AOR = 1.80, 95%CI: 1.17–2.78), having a mother who is a merchant (AOR = 1.96, 95%CI:1.09–3.51), and having a family member with a history of substance use (AOR = 2.93, 95%CI:2.02–4.24) were independently associated with substance use.

**Conclusion and recommendations:**

The overall magnitude of substance use among undergraduate students at Wallaga University was high. Factors such as sex, religion, monthly pocket money, department stream, mother’s occupation, and having a family member with a history of substance use were independently associated with substance use. Therefore, Wallaga University, along with its stakeholders such as the community around the campus area, students’ families, and NGOs working on substance use, should collaborate to tackle the problem effectively. Generally, substance use among students demands special attention; therefore, preventive measures and control strategies should be implemented to avoid substance use among students.

## Introduction

Substances are defined as any product (except food and water) that, when ingested, alters the way the mind (e.g., perception, consciousness, cognition mood, and emotions) and the body function (1). Substance use among university students is a major global public health issue and remains an important area of research due to the impact of substance dependence on the future of young people (2, 3). Previous research from various backgrounds has shown high levels of alcohol and other substance use among students in higher education (4). According to a survey conducted in Europe, 51.4% of the surveyed undergraduate university students consumed alcohol, 16.6% consumed both alcohol and cannabis, and 1.6% reported consuming alcohol and other illicit drugs (5).

The prevalence of substance use among undergraduate students in a developing country was 84.5%. Among these substances, alcohol exhibited the highest rate of lifetime use at 82.5%, with its 12-month prevalence accounting for 61.1%. There was a similar rate of lifetime use of psychoactive substances among male (86.1%) and female (83.4%) individuals (2). The studies conducted in Ethiopian higher learning institutions also showed high levels of substance use, especially alcohol, tobacco, and khat (6). Substance use, especially among university students, may lead to poor academic performance, which, in turn, can result in poor productivity in later life (7, 8). The use of alcohol, khat, and tobacco among adolescents can be harmful, leading to an increased risk of contracting HIV/AIDS and other sexually transmitted diseases (STDs), as well as multiple psychiatric disorders. In addition, substance use among undergraduate university students can lead to school dropout, strained relationship with peers, lack of interest in studying, poor academic performance, theft, and bullying (9). Furthermore, it exposes students to legal repercussions and may jeopardize their enrollment at the university (9).

Sociodemographic factors including sex, age, place of residence before joining the university, and year of study were significantly associated with current substance use (3, 10). Other factors related to a student’s family situation, such as parental employment status, frequent conflicts between parents, and the use of psychoactive substances by friends, roommates, or family members (brothers, sisters, or parents), were also significantly associated with substance use (2, 11). Campus lifestyle and environmental factors, including frustration and stress in the dorm and the availability and affordability of substances around campus, were other factors associated with substance use among undergraduate students (9).

Limited research on substance use and its associated factors has been conducted in our study area, with some studies conducted outside of it. Therefore, this study aimed to address the magnitude of substance use and its associated factors at Wallaga University.

Generally, the study result provides valuable information to policymakers, university administration, teachers, and other decision-makers on the prevalence of the problem and highlights the need for adopting better interventions and plans to address the issue.

## Objective

### General objective

The general objective of this study was to assess the magnitude and associated factors of substance use among undergraduate students at Wallaga University, Western Ethiopia, 2024.

### Specific objectives

1. One of the specific objective was to determine the magnitude of substance use among undergraduate students at Wallaga University, Western Ethiopia, 2024.
2. Another objective was to identify factors associated with substance use among undergraduate students at Wallaga University, Western Ethiopia, 2024.

## Materials and methods

### Study area and period

Wallaga University is located in the western part of Ethiopia and has three campuses: Nekemte Campus (Main Campus) located in East Wallaga Zone, Gimbi Campus located in West Wallaga Zone, and Shambu Campus located in Horro Guduru Wallaga Zone, Shambu town. Currently, 20,412 students are enrolled in undergraduate, postgraduate, and PhD programs. Of these, 5,913 regular undergraduate students attended classes across three campuses. Among these students, 4,124 were men and 1,789 were women. The study was conducted from 25 March 2024 to 2 May 2024.

### Study design

An institutional-based cross-sectional study design was employed.

### Source population

The source population included all undergraduate students studying in Wallaga University.

### Study population

The study population comprised all undergraduate students graduating from Wallaga University’s class of 2024, randomly selected from institutions/colleges/faculties of the university.

### Study unit

The study participants included all randomly selected undergraduate students from Wallaga University who provided complete data.

## Eligibility criteria

### Inclusion criteria

All undergraduate students who were studying for a bachelor’s degree during the study period were included.

### Exclusion criteria

Students who were on field trips at the time of data collection were excluded.

### Sample size and sampling technique

#### Sampling size determination

The sample size was calculated using the single population proportion formula, considering a 72.6% prevalence of substance use at Ambo University (9), a 5% margin of error, and a 10% estimated non-response rate.

$n=\frac{{\left(Z\alpha /2\right)}^{2}P\left(1-P\right)}{d^{2}}=\frac{{\left(1.96\right)}^{2}0.726\left(0.274\right)=306}{0.05^{2}}=306$

where n = required sample size.

P = prevalence.

d = margin of error.

Zα/2 = 1.96.

The sample size was calculated to be 306. To minimize errors arising from the likelihood of a non-response rate, 10% of the sample size, which was 31, was added to the initial sample, resulting in a total sample size of 337. Since a multistage sampling method was used, a design effect of 2 was applied, leading to a final sample size of 674.

The sample size for the second objective was calculated using Epi Info version 7 The parameters that were used to calculate the sample size were as follows: 95% confidence level (CI), 80% power, an unexposed-to-exposed group ratio of 1:1, and the selected variables, which included sex, stress/depression, and a family history of substance use (12, 13) (Table 1). Therefore, the sample size for factors associated with substance use was 328, which was the largest among the selected variables. Ultimately, the final sample size for this study was 674, as it was the largest compared to the other sample sizes.

**Table 1:** Sample size determination for associated factor of substance use among undergraduate students.

		Assumptions						Reference
Relevant factors	Power	Zα½	Percent among exposed	Percent among unexposed	Ratio	AOR	Sample size	
Sex	80	1.96	62	46	1	1.9	328	(15)
Stress/depression	80	1.96	80.7	55.7	1	3.30	124	(16)
Family history of substance use	80	1.96	95.6	60.5	1	0.05	52	(14)

##### Sampling procedure/technique

**Figure 1:**
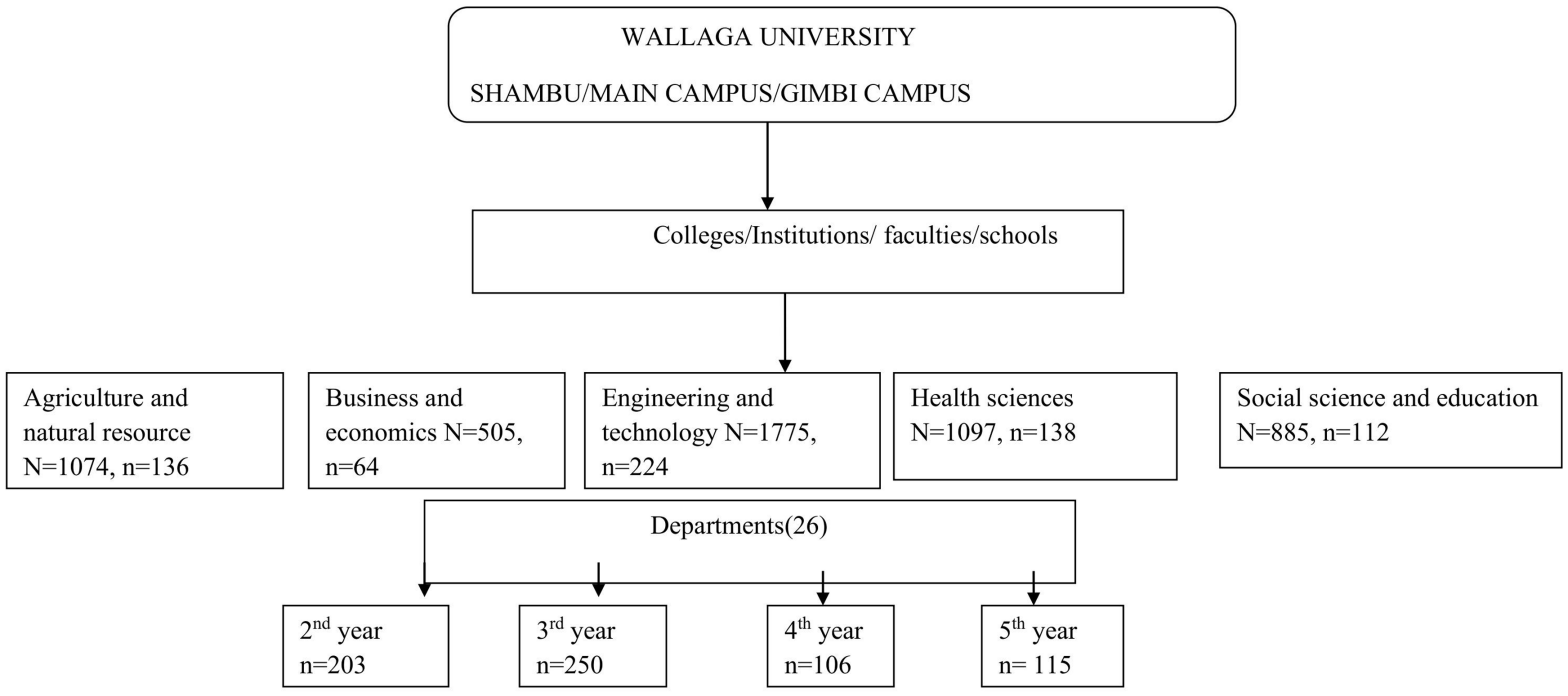
Schematic presentation of the sampling procedure employed to select participants from Wallaga University, 2024.

A multistage cluster sampling technique was used to select the study participants. Colleges, institutions, and faculties were randomly selected, followed by a random selection of departments. Further stratification was done based on the year of study. Finally, a simple random sampling technique was applied to select students from each year of study from the selected list of students, using their identification card numbers from their respective batches. During the study period year, first-year students were not present, so the study focused on students from years two to five. A schematic presentation of the sampling procedure is shown in Figure 1.

### Data collection instruments and procedure

The questionnaire was initially adapted after reviewing available scientific literature on similar studies and using the World Health Organization (WHO) (The Alcohol, Smoking and Substance Involvement Screening Test) ASSIST V3.0 tool, developed by the World Health Organization for use by all nations (14). This instrument (WHO ASSIST V3.0) is a questionnaire that is used for screening all levels of problem or risky substance use in adults. A standardized and self-administered questionnaire was prepared in English. The tool consists of three parts: Part I: Sociodemographic Characteristics, Part II: Current use of different substances, and Part III: Campus Lifestyle Questionnaire.

### Data quality control

One day of training was provided to the data collectors on the tool and objective of the study. The data collection tool was pre-tested on 5% (34) of the sample at Dambi Dollo University. Based on the findings and feedback obtained from the pre-test, no modifications were made, as the questionnaire was deemed appropriate and aligned with the objectives of the study. The pre-tested data were excluded from the final analysis. Supervisors were assigned during the data collection process.

### Data processing and analysis

The collected data were entered into EpiData version 4.6. Then, the data were exported to the Statistical Package for the Social Sciences (SPSS) for analysis. The data were analyzed using SPSS version 24, and descriptive analysis was used to describe the percentage and frequency distributions of the respondents’ sociodemographic characteristics. The mean and standard deviation were calculated for continuous variables, whereas frequency and proportion were calculated for categorical variables. The results were summarized and presented using tables and graphs. Bivariate and multivariable logistic regression analyses were performed to ascertain the association between explanatory variables and outcome variables. The associations were measured using adjusted odds ratios and their 95% confidence interval, and a *p*-value of less than 0.05 was considered statistically significant. The presence of multicollinearity was checked to control for the effects of potentially confounding variables using a multivariate logistic regression model, and the variance inflation factor (VIF) was found to be less than 1, which was acceptable.

#### Dependent variable

Substance use was the dependent variable.

#### Independent variables

The independent variables were age, sex, religion, family condition, year of study, department, monthly pocket money, family (mother and father) occupation, availability of substances, residential area before joining the university, peer pressure, campus lifestyle, health condition, and the types of high school and preparatory school attended.

#### Operational/term definition

- Substance use: This is defined as the use of any of the following substances—khat, alcohol, cigarettes, cannabis, and cocaine—for non-medical purposes (15).
- Current substance use: This is defined as the use of any of the substances listed above within the last 3 months (13)

## Results

### Sociodemographic characteristics

A total of 674 respondents participated, with a response rate of 94%. The mean age of the participants was 22.66 ± 2.21 years (SD), with ages ranging from 21 to 23 years for 382 (60%) respondents. The majority of the study participants, 439 (68.9%), were male. Nearly half of the study participants were Protestant (297, 46.6%), followed by Orthodox (215, 33.8%). Approximately half of the study participants, 323(50.7%), lived in urban areas before joining the university. A majority of participants were of Oromo ethnicity, comprising 404 (63.4%) individuals, followed by those belonging to Amhara ethnicity, comprising 135 (21.2%) individuals. Of the total study participants, 434 (68.9%) were from the natural science stream. The majority of the students’ fathers and mothers were farmers, accounting for 337 (52.9%) and 207 (32.5%), respectively. Moreover, 539 (84.6%) of the students’ parents were together, and 248 (38.9%) of the student’s family members had a history of substance use (Table 2).

**Table 2:** Socio economic and demographic characteristics of study participants of Wollega University regular undergraduate students’ of 2024 class.

Variables (*n* = 637)	Variables	Frequency (*n* = 637)	Percentage (%)
Age category	18–20	82	12.9
	21–23	382	60
	24–26	143	22.4
	>26	30	4.7
Sex	Male	439	68.9
	Female	198	31.1
Religion	Muslim	86	13.5
	Protestant	297	46.6
	Orthodox	215	33.8
	Catholic	8	1.3
	Wakefata	23	3.6
	Adventist	8	1.3
Residence before joining university	Urban	323	50.7
	Rural	314	49.3
Ethnicity	Oromo	404	63.4
	Amhara	135	21.2
	Tigray	17	2.7
	Wolayita	43	6.8
	Sidama	22	3.5
	Other^*^	16	2.5
marital status	Single	600	94.2
	Married	37	5.8
Where you attended high school	Public school	501	78.6
	Private school	136	21.4
Department by streaming	Natural sciences	434	68.9
	Social sciences	203	31.9
Father occupation	Employed^***^	188	29.5
	Merchant	100	15.7
	Farmer	337	52.9
	Others^**^	12	1.9
Mother occupation	Employed^***^	118	18.5
	Merchant	113	17.7
	Farmer	207	32.5
	Housewife	199	31.2
family member with history of substance use	Yes	248	38.9
	No	388	60.9
Current family marital status	Living together	539	84.6
	Divorced	43	6.8
	Mother or Father died	39	6.1
	Both died	2	0.3

Other^*^ Gambella, Silte, Guraghe, other ^**^ unemployed, laborer, employed^***^ Government and NGO workers.

### Year of the study

**Figure 2:**
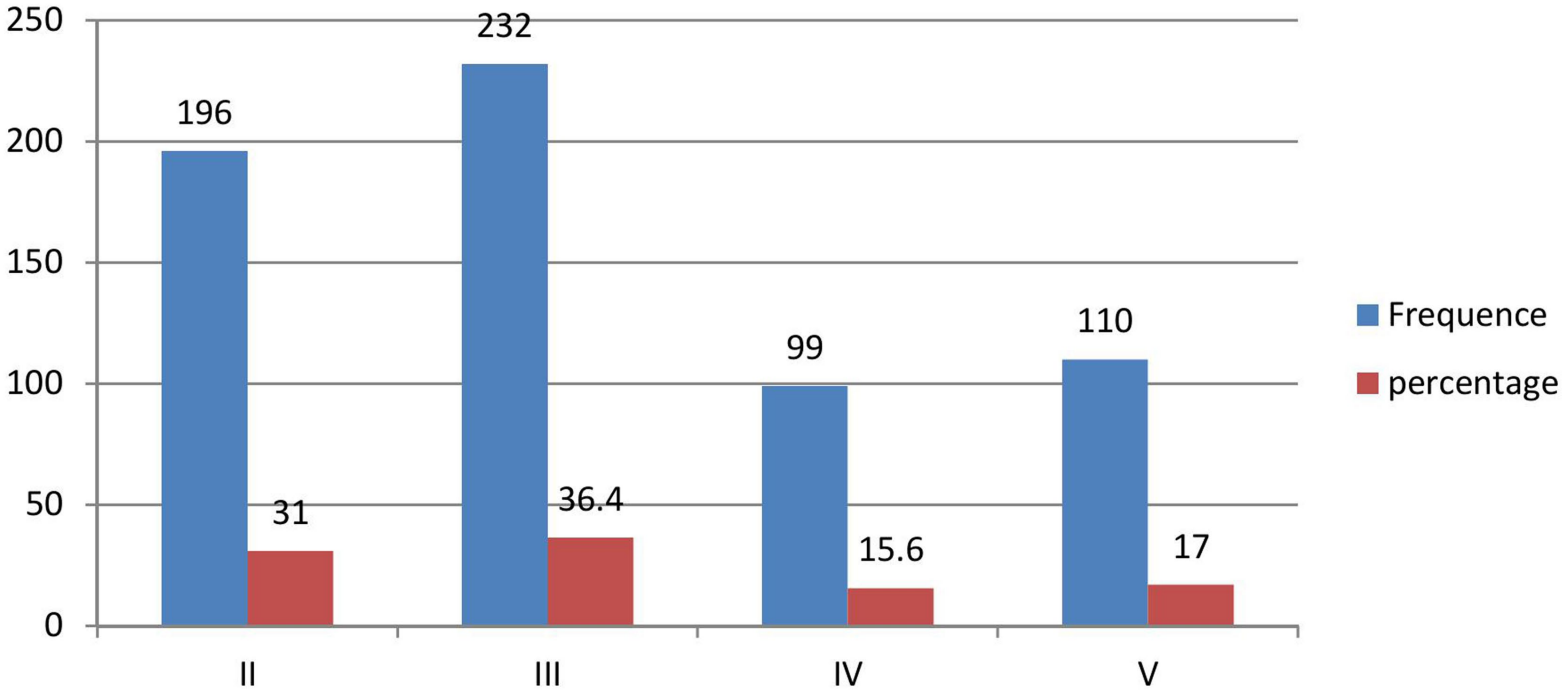
Year of study of the undergraduate students (*n* = 637) of the class of 2024 at Wallaga University.

Approximately 232 (36.4%) of the study participants were third-year students, followed by 196 (31%) second-year students (Figure 2).

### Monthly pocket money

**Figure 3:**
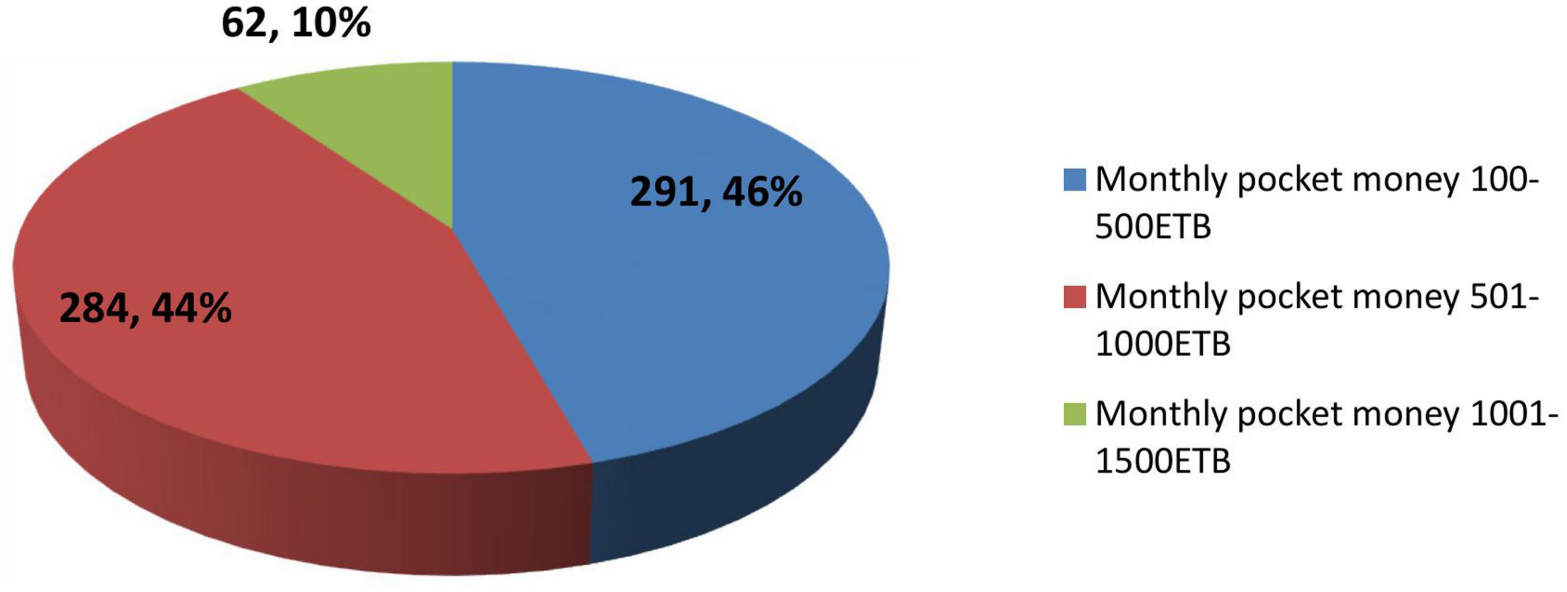
Pocket money of undergraduate students from the class of 2024 at Wallaga University (*n* = 637).

A total of 119 (46%) students had monthly pocket money ranging from 100 to 500 ETB, followed by 284 (44%) students with monthly pocket money between 501 and 1,000 ETB (Figure 3).

## Magnitude of substance use

The overall magnitude of current substance use among the y undergraduate students was reported in 188 participants (29.5, 95%CI: 25.96–33.04%), with a margin of error of ±3.542. The current use of alcohol, khat, and tobacco was reported by 145 (22.8%), 94 (14.8), and 16 (2.5%) participants, respectively. Classes of cannabis substances, such as ganja and shisha, accounted for approximately nine (1.4%) and eight (1.3%) participants, respectively (Table 3).

**Table 3:** Magnitude of substance use among Wollega University undergraduate, students, 2024.

Variables (*n* = 637)	Responses	Frequencies (*n*)	Percentage (%)
Any substances use within 3 months	**Yes**	**188**	**29.5**
	No	449	70.5
Khat	Yes	94	14.8
	No	543	85.2
Alcohol	Yes	145	22.7
	No	492	77.3
Cigarette	Yes	16	2.5
	No	621	97.5
Shisha	Yes	9	1.4
	No	628	98.6
Ganja	Yes	8	1.3
	No	629	98.7

Bold values indecate magnitude of substance use.

### Campus lifestyle and substance use

A total of 122 (64.9%) students with current substance use had friends with a history of substance use, and 104 (78.8%) of them tried substances because of their peers’ influence. In addition, 110 (58.5%) students used substances because they believed it alerted their minds (Table 4).

**Table 4:** Campus life style and substance use among Wollega University undergraduate students, 2024.

Variables (*n* = 188)	Response	Frequency	Percent
Friends with substance use	Yes	122	64.9
	No	66	35.1
Peer pressure	Yes	104	78.8
	No	84	44.6
Any academic dissatisfaction?	Yes	55	29.3
	No	133	70.7
Substance use for being alert	Yes	110	58.5
	No	78	41.5
easily availability of substances around university	Yes	121	64.4
	No	67	35.6
Easily Afford these substances	Yes	96	51.1
	No	92	48.9
Do you mostly feel stressed?	Yes	43	22.9
	No	145	77.1
Have chronic medical conditions?	Yes	7	3.7
	No	181	96.3
Have known mental illness?	Yes	5	2.6
	No	183	97.4

### Factors associated with substance use among the undergraduate students at Wallaga University

A bivariate logistic regression analysis was conducted, and variables with a *p*-value of <0.25 were considered candidates for a multivariable analysis. Variables such as sex, pocket money, department stream, mother’s occupation, and having a family member with a history of substance use were independently associated with substance use in the final model. Male students were more likely to be substance users compared to the female students (AOR =1.95, 95%CI: 1.27–2.78). The students whose monthly pocket money was between 1,001 and 1,500 ETB were 2.27 times more likely to be current substance users compared to those whose monthly pocket money was <1,000 ETB (AOR = 2.27, 95%CI: 1.20–4.28). Students from the natural science stream were 1.80 times more likely to be current users compared to those from the social science stream (AOR = 1.80, 95%CI: 1.17–2.78). Finally, students with family members who use substances were 2.93 times more likely to use substances compared to those whose family members never use substances (AOR = 2.93, 95%CI:2.02–4.24) (Table 5).

**Table 5:** Multivariable analysis of factors affecting substance use among Wollega university undergraduate students, 2024.

Variables	Response	Substance use		COR (95% CI)	AOR (95% CI)	*p* value
		Yes	No			
Sex	Male	144	295	1.7 (CI:1.16–2.52)	1.95 (95%CI:1.27–2.78)	0.002*
	Female	44	154	1	1	
Pocket money	100-500ETB	70	221	1	1	
	501-1000ETB	88	196	1.41 (CI:1.19–3.65)	1.81 (95%CI:0.97–3.40)	0.063
	1,001-1500ETB	30	32	2.96 (CI: 1.68–5.2)	2.27 (95%CI:1.20–4.28)	0.012*
Department streaming	Natural science	144	290	1.79 (CI:1.27–2.65)	1.80 (95%CI:1.17–2.78)	0.008*
	Social sciences	44	159	1	1	
Father occupation	Employed	68	119	0.99 (CI:0.6–1.63)	0.996 (95%CI:0.56–1.8)	0.99
	Merchant	37	64	1.81 (CI:1.2–2.7)	1.3 (95%CI:0.72–2.5)	0.413
	Farmer	81	256	1	1	
	Other	2	10	2.9 (CI:0.61–13.43)	2.9 (95%CI:0.56–14.77)	0.206
Mothers’ occupation	Employed	88	73	1.05 (CI:0.61–1.81)	0.91 (CI:0.46–1.66)	0.75
	Merchant	42	71	2.04 (CI:1.24–3.38)	1.96 (CI:1.09–3.51)	0.024*
	Farmer	47	160	1	1	
	House wife	54	145	1.61 (CI:0.98–2.64)	1.79 (CI:1.02–3.13)	0.041*
Family member with history of substance use	Yes	109	139	3.067 (CI:2.16–4.36)	2.93 (95%CI:2.02–4.24)	<0.001*
	No	79	309	1		

1 = Referent and *Significant association.

## Discussion

The aim of this study was to assess the magnitude of substance use and its associated factors among undergraduate students at Wallaga University in Western Ethiopia. The study revealed that the overall magnitude of substance use among undergraduate students was 29.5%, with a 95%CI of 25.96–33.04%. This finding is in line with that of a study conducted at Haramaya University, which reported a current substance use rate of 32% but differed in the type of substances consumed (11). Khat was the most consumed substance in that study, while alcohol was the most consumed substance in our study. This difference could be attributed to khat being easily available and affordable at Haramaya University, and in our study area, this substance is very expensive. Another reason could be differences in sample size, as the sample size in our study area was three times larger than the sample size of the study conducted at Haramaya University.

However, this result was greater than those of studies conducted at Debre Birhan University and Mekelle University (16, 17). The current use of alcohol, khat, and cigarettes at Debre Birhan University was approximately 16.9, 5.7, and 3.1%, respectively, while in our study area, it was 22.8, 14.8, and 2.5%, respectively. This difference could be attributed to differences in methodology and the sociodemographic characteristics of the study participants. In addition, this study’s result was also higher than that of a study conducted at Mekelle University, which reported 21.03%. In terms of substance use in the last 3 months, 3% of the participants were khat users, 12% were alcohol drinkers, and 4.8% were cigarette smokers. The difference could be attributed to the geographical location of the study area, the sample size, which was twice as large as that of the study conducted at Mekelle University, and the fact that the study at Mekelle University included only health college students (17).

This result was lower than those of studies conducted at Ambo University, Adama University, and Adigrat University (8–10). The study conducted at Ambo University indicated that the prevalence of current substance use was 72.6%, which was greater than that of this study area (9). This finding might be due to the fact that students studying at Awaro campus of Ambo university were from a single college which was college of technology; and did not consider social science stream or other natural science stream/college.

The prevalence of substance use at Adama Science and Technology University (ASTU) was 55.2%, with the most commonly used substances presented in descending order: alcohol (51.7%), khat (23.7%), and cigarettes (12.2%). The prevalence found in the previous study was higher than the prevalence found in this study (10). The gap may be attributed to the fact that the study at ASTU included only the graduating class.

This study also indicated that the students used substances due to peer pressure, academic dissatisfaction, and the need to stay alert during studies, as well as because they were easily accessible and affordable. This finding is in line with that of a study conducted at a university in Nigeria, where peer pressure was also a reason why most students started using substances (12, 18). It can be observed that the reasons for substance consumption vary from one country to another, emphasizing that cultural, social, and economic factors can have a direct influence on consumption (19). This study also assessed the duration of substance use initiation, and most of the students started using substances 4 years ago or more. This result is in line with that of a study conducted at Haramaya University. According to the study, nearly half of the students started using substances more than 4 years ago (11).

The study revealed that sex, monthly pocket money, department stream, mother’s occupation, and having a family member with a history of substance use were independently associated with substance use. Being male was also associated with substance use. This finding is consistent with those of studies conducted at Ambo University, ASTU, and Haramaya University (9–11). Substance use in male individuals was reported to be 1.95 times higher compared to that in female students (AOR = 1.95, 95%CI, (1.27–2.78)). This difference might be because male students are more exposed to substances and peer pressure is more common among them than among female students. Moreover, many substances such as khat, tobacco, and alcohol are socially accepted when used by male individuals. The family, the university, and the community should work together to address this misconception of substance use among male students.

The students from families with a history of substance use were 2.93 times more likely to use substances compared to other students (AOR = 2.93, 95%CI, 2.02–4.24). This finding is supported by previous studies performed at Debre Birhan University, Addis Ababa University (AAU), and others (16, 20), which might be because many instances of first-time substance use occur in family settings and under parental supervision. In addition, the decision of parents to allow their children to use substances may be influenced by a family history of substance use. Therefore, parents should be cautious when using substances in front of their children.

Furthermore, department stream was also a factor in substance use among undergraduate students. Students in the natural science stream were more likely to use substances than those in the social science stream (AOR = 1.72, 95%CI, (1.11–2.65)). This finding might be due to the burden of courses mostly lie on natural science students as compared to social science students.

In addition, the mother’s occupation was also associated with the use of substances among undergraduate students. Students with merchant mothers were 1.96 times more likely to use substances compared to students whose mothers were farmers (AOR = 1.96, 95%CI, (1.09–3.51)). This might be because mothers are passionate about supporting their children, and merchant mothers tend to have more liquid money compared to farmer mothers. Therefore, in addition to the money given to children by their fathers, mothers may hide money from their husbands and send it to their children. This finding makes substances more easily affordable to students who have more money. Mothers should be cautious about the money they send to their children and refrain from encouraging extravagant lifestyles on campus.

Finally, receiving monthly pocket money of more than 1,000 ETB was significantly associated with substance use at Wallaga University. Substance use among those who receive more than 1,000 ETB was 2.27 times higher compared to those receiving <1,000 ETB (AOR = 2.27, 95%CI, (1.20–4.28)). This result is consistent with those of studies conducted in Kenya, AAU, Ambo University, and Adigrat University (8, 9, 13, 19, 20). This finding implies that students who receive more pocket money are more likely to use substances compared to those who receive relatively less. Consequently, regulating the amount of pocket money can be one of the steps to address the issue of substance use. There is also a need to educate students on how to manage and utilize their pocket money wisely so that it is not spent on harmful uses.

## Conclusion and recommendations

This study assessed the overall magnitude of substance use among undergraduate students at Wallaga University. Factors such as sex, pocket money, department stream, mother’s occupation, and having a family member with a history of substance use were independently associated with substance use among undergraduate students.

Therefore, Wallaga University, along with stakeholders such as the community around the campus area, students’ families, and NGOs working on substance use, should collaborate to tackle the problem. Generally, substance use among students demands special attention, and preventive measures and control strategies, including awareness campaigns on the potential impacts of substance use, should be implemented.

## Limitations of the study

As this study was cross-sectional in nature, it only focused on quantitative results and did not explore the life experiences of the students who use substance. Therefore, we recommend that future research should consider a mixed-methods approach.

## Data Availability

The raw data supporting the conclusions of this article will be made available by the authors, without undue reservation.
